# Microenvironment Elements Involved in the Development of Pancreatic Cancer Tumor

**DOI:** 10.1155/2012/585674

**Published:** 2012-12-13

**Authors:** Katarzyna Gardian, Sława Janczewska, Marek Durlik

**Affiliations:** ^1^Department of Surgical Research and Transplantology, Mossakowski Medical Research Centre, Polish Academy of Sciences, 5 Pawinskiego Street, 02-106 Warsaw, Poland; ^2^Department of Gastroenterology and Transplantology Surgery, Central Clinical Hospital of the Ministry of the Internal Affairs in Warsaw, Poland

## Abstract

*Introduction*. In spite of intensive research during many years, pancreatic adenocarcinoma remains one of the deadliest cancers. The surgical intervention remains main possibility of treatment because chemotherapy and radiotherapy has a minimal impact on long-term survival. We are still looking for the weak points of this devastating disease. *Materials and Methods*. Pancreatic tumor tissue samples were collected from 36 patients. Immunohistochemistry staining was used to evaluate expression of growth factors and immune infiltrates. Activity of MMP2 and MMP9 was assessed by gelatin zymography on 7.5% SDS-PAGE gel with 0.1% gelatin. *Results*. All growth factors were strongly expressed in pancreatic tumor tissue. We found that level of expression of c-Met receptor was higher for G3 tumors than for G2 tumors. Also we found that active MMP2 was present at all stages of tumor while active MMP9 just at more advanced tumors. Abundant immune cells infiltration was distinctive for tumor tissue, especially macrophages were infiltrating tumor tissue. We found that amount of macrophages was associated with lymph nodes metastases. *Conclusion*. In our research we demonstrated that among many factors influencing tumor microenvironment c-Met receptor, infiltrating macrophages and MMP2 have significant influence on development and invasion of pancreatic cancer.

## 1. Background

In spite of intensive research during many years, pancreatic adenocarcinoma remains one of the deadliest cancers. The surgical intervention remains main possibility of treatment because chemotherapy and radiotherapy has a minimal impact on long-term survival. Research over the last twenty years has yielded much insight into pancreatic cancer biology, but it has neither improved diagnostics methods nor the way of treatment. We are still looking for the weak points of this devastating disease.

It was shown in recent years that the tumor microenvironment plays a critical role in tumor progression [1]. In pancreatic tumors this microenvironment is particularly heterogeneous. It consists of dense fibrotic stroma with cancer cells, stellate cells, infiltrating inflammatory cells, and the remains of the proper structure of the pancreas. These cells are the source of various growth factors as well as proangiogenic factors. The fibroblasts present in the tumor's tissue are responsible for the production of collagen and fibronectin which increase the chemoresistance of the tumor. Because of abundant fibrotic tissue, the tumor environment is strongly hypoxic [2, 3]. All these factors contribute to the disease's aggressive nature and occurrence of early metastases.

Infiltrating inflammatory cells are a rich source of factors influencing tumor growth, invasion, and metastases. Macrophages are of particular interest. Their role in the tumor's environment has been studied in recent years [4]. It is known that they are arich source of growth factors that stimulate cell proliferation like EGF, PDGF-BB, HGF*α*, and TGF *β*. Furthermore they also produce matrix metalloproteinase-9 (MMP9) which takes part in various essential processes [5].

Matrix metalloproteinases are a group of 20 proteases divided into 4 subclasses: collagenases, gelatinases, stromelysins, and membrane-type MMPs. They take part in modification of extracellular matrix which makes them important component of such processes as angiogenesis, cell migration and metastasis formation. They also participate in activation of many proteins and in this way they can regulate proliferation and apoptosis [6]. 

MMP 2 and 9 are classified as gelatinases. Their role in metastasis formation is significant because their substrate is collagen IV, a component of basement membrane. Its degradation makes it possible to migrate for cancer cells. Increased expression of MMP 2 and 9 was stated in many types of cancer. In breast cancer [7] their activity was associated with distant metastasis and in case of oesophageal carcinoma with increases in invasiveness [8]. 

Also in pancreatic cancer strong expression of MMPs was shown. In a research conducted on 6 cell lines, it was shown that increased expression and activity of MMP2 are associated with greater invasive potential of pancreatic cancer cells [9]. 

The aim of our study was to investigate three points that may have an influence on the development of the tumor: expression of growth factors, inflammatory cells infiltration, and enzymatic activity of matrix metalloproteinases so as to have a more complete picture of the pancreatic cancer tumor. 

## 2. Materials and Method

### 2.1. Patients and Specimen Collection

Pancreatic tumor tissue samples were collected from 36 patients who underwent surgical resection due to pancreatic cancer. Tissues were collected based on protocol approved by the Bioethics Committee of Warsaw Medical University. Tumors were classified according to TNM staging and tumor grade. PDAC patients ranged between T1–T4 (T1 (*n* = 3), T2 (*n* = 6), T3 (*n* = 25), and T4 (*n* = 2)), N0 (*n* = 13), N1 (*n* = 23), M0 (*n* = 33), and M1 (*n* = 3) stage. Also the histologic grade was evaluated: G1 (*n* = 4), G2 (*n* = 16), and G3 (*n* = 16). 

For protein isolation, the samples were frozen and stored at −20°C until they were used. Samples for immunohistochemical analysis with dimensions of 5 × 5 × 5 mm were frozen for 45 seconds in acetone using dry ice at a temperature of −70°C and stored at −80°C.

### 2.2. Gel Zymography

Proteins were isolated from tumor tissue using Total Protein Extraction Kit (Millipore, Billerica, USA). 10 *μ*g of total protein isolated from the tissue was diluted with 2 parts of Zymogram sample buffer (Bio-Rad Laboratories, Hercules, USA) and resolved by 7.5% SDS-PAGE containing 0.1% gelatin (Sigma-Aldrich, St. Louis, USA). Following electrophoresis, gels were washed with 2.5% Triton X-100 to remove SDS twice for 30 minutes and incubated in developing buffer (50 mM pH 7.5 TRIS buffer with 5 mM CaCl_2_ and 0.2 M NaCl) for 20 h at 37°C. Gelatinase activity was visualized by staining the gels with 0.5% Comassie blue for 1 h followed by incubation in the destaining solution (methanol 40%, acetic acid 10%). Afterwards the destained gel was rinsed with water. For semiquantitative evaluation, a photo of gel was taken and MicroImage (Olympus, Japan) software was used.

### 2.3. Immunohistochemistry

Frozen tissue from pancreatic cancer was cryocut into 5 *μ*m sections. Each tissue was stained with hematoxylin-eosin (H&E). The Dako REAL EnVision Detection System, Peroxidase/DAB+, Rabbit/Mouse were used for immunostaining. After being dried in room temperature, the slides were fixed with acetone for 10 min. Then they were incubated for 5 min Dual Endogenous Enzyme Block (Dako, Glostrup, Denmark). The sections were incubated with a proper antibody for 25 minutes for EGF (Z-19), EGFR (EGF-R2), PDGF-BB (N-30), HGF*α* (H-145), and c-Met (C-12) (all form Santa Cruz Biotechnology, Santa Cruz, USA) and for CD68 and CD3 (EBM11; F7.2.38, Dako, Glostrup, Denmark). Afterwards incubation with Dako REAL EnVision/HRP, Rabbit/Mouse (ENV) for 25 minutes at room temperature was followed by a color reaction using Dako REAL DAB+ chromogen for 3 minutes. The slides were counterstained with Mayer's hematoxylin. 

### 2.4. Semi-Quantitative Analysis of Immunohistochemical Staining

For quantitative evaluation, 5 areas were chosen after scanning the tumors sections at low power 40x. These fields were analyzed at 200x magnification using MicroImage software (Olympus, Japan), counting the total stained area. 

### 2.5. Statistical Analysis

A comparison was made: for two groups with the Mann-Whitney *U* test and for three groups with Kruskal-Wallis Test. The minimal level of significance was defined as *P* < 0.05.

## 3. Results

### 3.1. Growth Factors in Pancreatic Cancer

**Figure 1:**
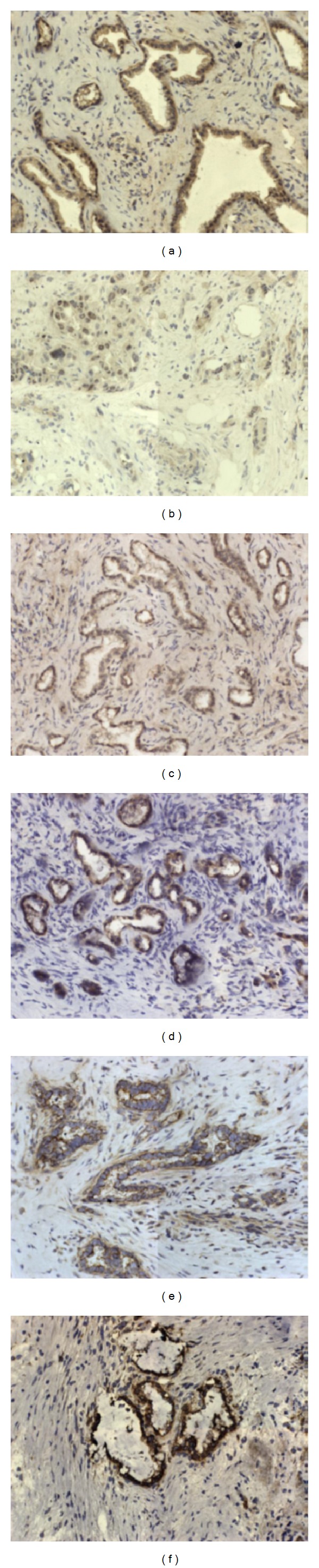
Expression of growth factors: (a) PDGF-BB cancer nests, (b) PDGF-BB stroma, (c) EGF, (d) EGFR, (e) HGF*α*, (f) c-Met. Original magnification ×200.

25 tumor tissues were studied immunohistochemically for expression of growth factors in tumor tissue (Figure 1). Expression of growth factors was found in all cases. The immunoreactivity of EGF was weak to moderate in cytoplasm of cancer cells. As for EGFR we found its expression to be moderate to strong in cytoplasm of cancer cells and weak in small ductal cells. PDGF-BB immunoreactivity was moderate to strong in cytoplasm of cancer cells and also in 6 cases we found nuclear staining in cancer cells as well as in infiltrating immune cells. Membranous and cytoplasmic staining for HGF*α* was strong in tumor cells whereas staining of c-Met was moderate to strong.

**Figure 2:**
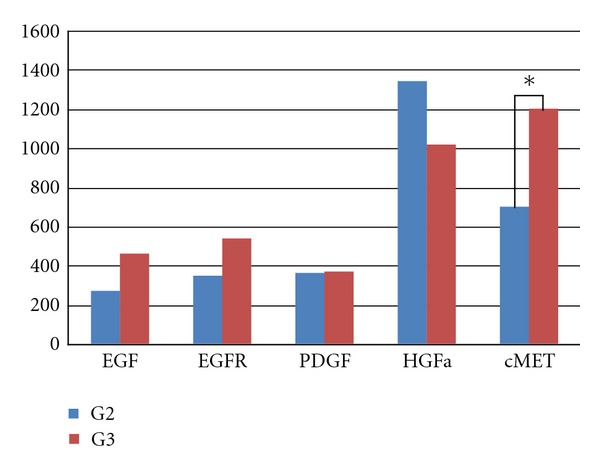
Comparison of expression of growth factors idn G2 and G3 tumors.

Expression of growth factors was compared against tumor grading and N stage. Using a semiquantitative estimation of all considered growth factors expression, the result of statistical analysis showed that the only difference in expression between G2 and G3 group was statistically relevant in case of c-Met receptor (*P* = 0.033) (Figure 2). 

### 3.2. Infiltrating Inflammatory Cells

**Figure 3:**
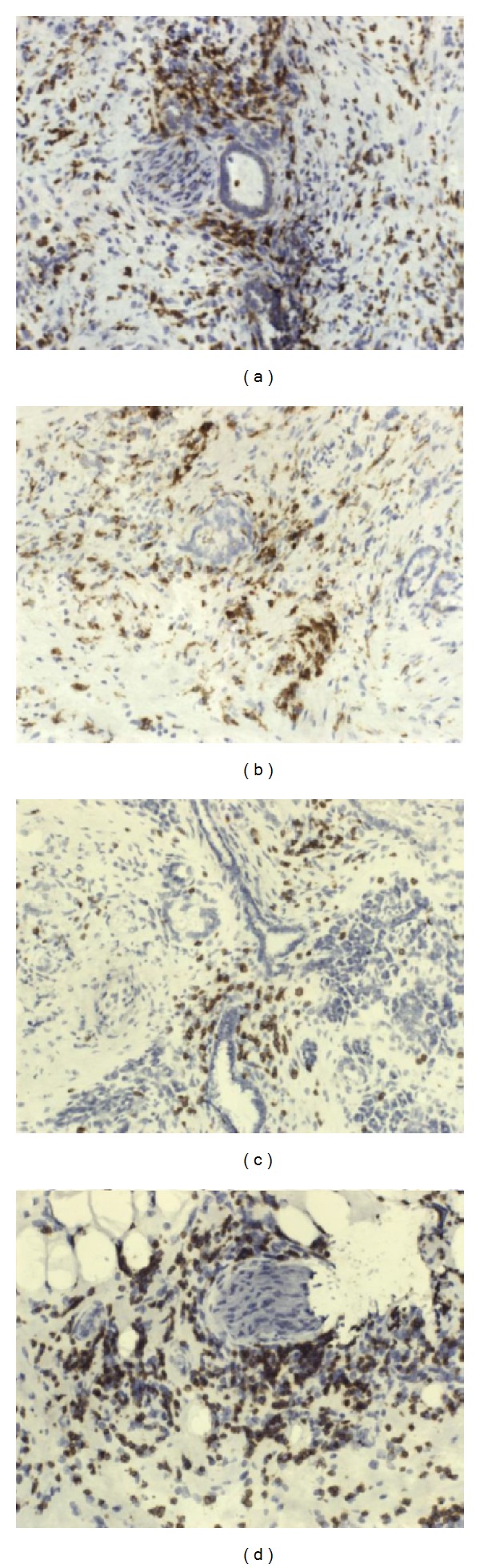
Characterization of the inflammatory infiltrate (a) and (b) CD68 macrophages (c) and (d) CD3: original magnification ×200.

To evaluate cell infiltrates we used monoclonal antibodies against CD68, HLA II, neutrophil elastase, CD3, and CD56. We found numerous lymphocyte and macrophages infiltrations. There was also a strong expression of neutrophil elastase. No NK cells infiltration was observed. Inflammatory cells were present around neoplastic glands and also strongly around nerves infiltrated by cancer cells (Figure 3). 

**Figure 4:**
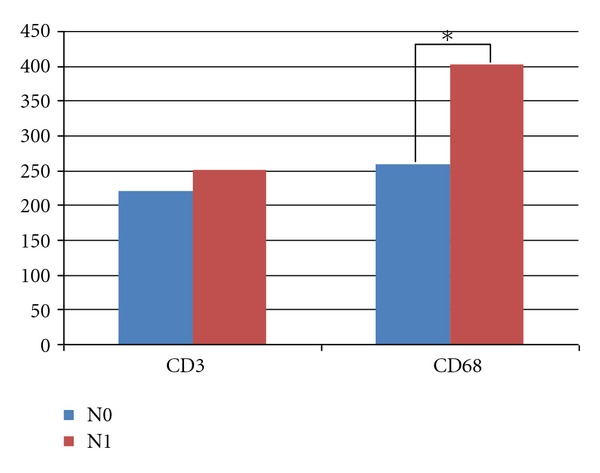
Comparison of inflammatory infiltrates.

We compared the results due to N stage and we found that the number of macrophages in tumor tissue was significantly higher in the group with metastases to lymph nodes (401) than the in N0 group (167) (*P* = 0.0085) (Figure 4).

### 3.3. Gel Zymography

Genolytic activity was studied in 30 tissue isolates from pancreatic tumors. Active MMP2 (62 kDa) was present in 88% cases and MMP9 (83 kDa) in 38% cases. For 6 samples we were not able to determine MMP's activity because of indistinct picture of gel.

Comparing the results according to histologic trading we can tell that for G1 tumors we did not observe activity of matrix metalloproteinase 9. For G2 tumors active MMP9 was present in 7 (*n* = 9) cases and for G3 only for 4 (*n* = 11). Appearance of active MMP2 was claimed for 3 G1 cases (*n* = 4), in all samples for G2 and for 10 G3 cases (z 11).

Densitometric measurement also confirmed that for well-differentiated tumors matrix metalloproteinases' activity is lower than for G2 and G3 (*P* < 0.05). Activity of MMP2 was, respectively, G1: 3.27 ± 3.6; G2: 16.57 ± 13.9; G3: 13.6 ± 12.2 (*P* < 0.05). Activity of MMP9 was not reported for G1 tumors, and for the other groups it was, respectively, G2: 18 ± 13.9; G3:  38.2 ± 22.3.

## 4. Discussion

In pancreatic tumors we observed intensive immune cells infiltration. In pancreatic cancer it was reported previously that macrophages are involved in angiogenesis [10], supporting tumor growth and invasion of cancer cells. They are the source of angiogenic factors like VEGF and also MMP9 which degrade extracellular matrix. Tdhe important fact is that macrophages can suppress T cell response. Thus, macrophages infiltrating pancreatic tumor are an important factor in creating metastases. We found that the number of macrophages is higher in the group with lymph node metastases. This observation supports the statement about participation of macrophages in creating metastases.

Abundant expression of growth factors is typical for pancreatic cancer tissue. As they participate in signaling pathways mediated by receptor tyrosine kinases, they might regulate cell proliferation, migration, and survival. Previous studies on expression of growth factors provided and interesting observation. It was reported that a high level of EGF and EGFR correlated with lymph node involvement and distant metastasis as well as reduced median survival [11]. Only in case of PDGF-BB high levels in blood serum were favorable in prognosis [12] and high expression in tumor was related to decreased pancreatic cancer growth [13]. In our research we observed a strong expression of growth factors; however, only for c-Met receptor we were able to claim a statistically significant difference between G2 and G3 groups. No significant difference was observed in relation to lymph node metastases.

Our results indicate that c-Met might be a pivotal element in pancreatic cancer. This receptor tyrosine kinase is activated by HGF and physiologically it participates in embryonic development and also during adult life in liver regeneration and wound healing [14]. However its overexpression is observed in pancreatic cancer, even in the early stage of carcinogenesis [15]. 

c-Met activation leads to increased proliferation, enhanced motility, and invasion. Therefore we presume that an increase in c-Met expression between G2 and G3 stages indicates a more aggressive phenotype which might be related to epithelial-mesenchymal transition. EMT is often considered a first step to metastases as it is related to reduced E-cadherin expression and appearance on N-cadherin [16].

 Another factor was linked to lymph nodes involvement—presence of macrophages—this was also confirmed by our studies. Our results indicate that the amount of macrophages is significantly higher in the group with lymph node metastases, as it was reported previously [17]. Recently it has been shown that macrophages also participate in EMT process [18]. They not only express EMT-inducing cytokines but also MMP9 that cleaves E-cadherin/*β*-catenin complex. Tan et al. suggested that macrophage MMP9 supports EMT also by disrupting of basement membrane. This is a crucial mechanism for invasion of cancer cells to other tissues.

Our analysis of activity of MMPs indicates that MMP2 has a greater impact on development of pancreatic cancer. Its activity can be observed even in the early stages of tumor. On the contrary activity of MMP9 was observed only in G2 and G3 groups. MMP2 was previously reported to increase ability of cancer cells to migrate [19] and determination of MMP2 activity in pancreatic juice was told to be useful in diagnosing pancreatic cancer [20]. MMP9 was associated with metastasis and angiogenesis [21]. Lack of its activity in G1 group might indicate that it is triggered in subsequent stage of the tumor.

## 5. Conclusion

In our research we demonstrated that among many factors influencing tumor microenvironment c-Met receptor, infiltrating macrophagesو and MMP2 have a significant influence on development and invasion of pancreatic cancer. They all might contribute to EMT and we intend to examine this in our further research.
